# Selection of Breeding Stock among Australian Purebred Dog Breeders, with Particular Emphasis on the Dam

**DOI:** 10.3390/ani6110075

**Published:** 2016-11-16

**Authors:** Veronika Czerwinski, Michelle McArthur, Bradley Smith, Philip Hynd, Susan Hazel

**Affiliations:** 1 School of Animal and Veterinary Sciences, The University of Adelaide, Mudla Wirra Rd, Roseworthy, SA 5371, Australia; michelle.mcarthur@adelaide.edu.au (M.M.); philip.hynd@adelaide.edu.au (P.H.); susan.hazel@adelaide.edu.au (S.H.); 2 School of Human, Health and Social Sciences (Appleton Institute), Central Queensland University, 44 Greenhill Road, Wayville, SA 5034, Australia; b.p.smith@cqu.edu.au

**Keywords:** dog breeding, purebred dog, survey, maternal care

## Abstract

**Simple Summary:**

One of the most important factors influencing the health and welfare of puppies is the decision made by the breeder on which dam and sire they will breed from. Unfortunately, our understanding of what dog breeders consider important when selecting their dogs, particularly the dam, is limited. In order to bridge this gap, we conducted an online survey of Australian purebred dog breeders. We identified four major factors that the breeder considered important in relation to the dam: Maternal Care; Offspring Potential; Dam Temperament; and Dam Genetics and Health. Overall, the priorities and practices of dog breeders surveyed were variable across breeds. Importantly, it seemed that not all breeders understood the importance of maternal care behaviour, despite the significant role it may play on future puppy behaviour.

**Abstract:**

Every year, thousands of purebred domestic dogs are bred by registered dog breeders. Yet, little is known about the rearing environment of these dogs, or the attitudes and priorities surrounding breeding practices of these dog breeders. The objective of this study was to explore some of the factors that dog breeders consider important for stock selection, with a particular emphasis on issues relating to the dam. Two-hundred and seventy-four Australian purebred dog breeders, covering 91 breeds across all Australian National Kennel Club breed groups, completed an online survey relating to breeding practices. Most breeders surveyed (76%) reported specialising in one breed of dog, the median number of dogs and bitches per breeder was two and three respectively, and most breeders bred two litters or less a year. We identified four components, relating to the dam, that were considered important to breeders. These were defined as Maternal Care, Offspring Potential, Dam Temperament, and Dam Genetics and Health. Overall, differences were observed in attitudes and beliefs across these components, showing that there is variation according to breed/breed groups. In particular, the importance of Maternal Care varied according to dog breed group. Breeders of brachycephalic breeds tended to differ the most in relation to Offspring Potential and Dam Genetics and Health. The number of breeding dogs/bitches influenced breeding priority, especially in relation to Dam Temperament, however no effect was found relating to the number of puppies bred each year. Only 24% of breeders used their own sire for breeding. The finding that some breeders did not test for diseases relevant to their breed, such as hip dysplasia in Labrador Retrievers and German Shepherds, provides important information on the need to educate some breeders, and also buyers of purebred puppies, that screening for significant diseases should occur. Further research into the selection of breeding dams and sires will inform future strategies to improve the health and behaviour of our best friend.

## 1. Introduction

There are an estimated 4.2 million dogs within Australia [1] with most dogs bred for the purpose of companionship [2]. In Australia, the Australian National Kennel Club (ANKC) is the registered organisation for pedigree dog breeders. In 2015, its 32,481 members, 20% of whom were active breeders, produced (and registered) 69,274 puppies [3]. Given that it is impossible to determine the number of puppies born to non-registered breeders, the total number of puppies born in Australia each year is unknown.

One of the primary aims of the ANKC is to provide members with breed standards that promote behaviourally and physically sound dogs for ownership, as well as promoting excellence in a number of dog-related fields, such as breeding, showing, trialling, obedience and other canine related behaviour. Although the ANKC collects information about registered breeders (i.e., number of breeders per breed, number of active breeders, number of litters produced per year, number of puppies produced) through the state bodies, data collected does not extend to the breeding priorities and practices of the breeders. This lack of information extends to the scientific literature, with little known about dog breeding practices and philosophies in Australia for registered and non-registered breeders. Such information is vital for improving breeding practices, and ensuring the optimal health and behaviour of dogs.

With over 200 breeds registered with the ANKC, breeding practices are likely to be as diverse as the breeds themselves [4]. For example, the purpose for which the dog is bred (i.e., companion, working) is likely to be reflected in the way the dogs are housed and bred. Often, the most important aspect of pedigree or pure breeding involves the selection of breeding animals that conform to a set standard [5], which is usually determined by a registered organisation such as the ANKC. Physical characteristics (e.g., body conformation, coat length and colour, height, facial appearance, gait), as well as certain behaviours (e.g., instincts such as herding, hunting or retrieving, temperament and trainability) are taken into consideration when choosing breeding stock [6]. Priorities of breeders are also likely to alter over time. For instance, in the past, dogs were primarily bred for various working roles, but the focus has shifted to selecting for suitable companion animals, moving towards dog conformation rather than performance [5]. Breed specific diseases are now highly recognised [7,8], allowing for accessible knowledge to be implemented by the breeder. Health risks are also being associated with natural mating, and thus sire selection and mating techniques are also necessary to consider [9,10].

To date, the goals and practices of dog breeders across the world have received little attention. Notable exceptions include a study looking at inbreeding and breed effective population size in an Australian sample of breeders [11], and the selection of dogs and breed goals documented in a French population [4]. In that study [4], 985 French dog breeders, representing 10 different breed groups were asked what considerations they gave to conformation, behaviour, health, work, feeling and reproduction. The behaviour of the dog was considered significantly more important by breeders of sheepdogs, cattle dogs and retrievers compared to all other dog groups. Although the number of litters produced did not significantly alter breed goal, litter production was impacted by breed group; working dogs produced less litters than other breed groups [4]. Leroy et al. [4] also discovered that there were different types of breeders (i.e., occasional, regular hobby and professional breeders) and regular hobby and professional breeders bred from their bitches earlier and therefore had more litters throughout the dam’s life. Overall, breeders reported four common goals: (1) dog conformation; (2) behaviour; (3) health and (4) work. Notably, breeders did not consider maternal care as a factor in the selection of breeding bitches [4], despite the importance that it can have on offspring development (e.g., [12,13,14]). Other factors including the type of birth (i.e., natural vs caesarean), may also affect the dam’s behaviour towards the puppies. Caesareans are more likely to occur in certain breeds according to their cranial features [15,16,17], yet there is no literature on the impact of birth type and maternal behaviour.

Three recent studies have highlighted the importance of maternal care in dog development [18,19,20]. A correlation was found between maternal care and later anxiety in puppies, with poor maternal care in puppyhood increasing the likelihood of anxiety in dogs, measured using questionnaires [18]. In the second study, maternal care (dam in box, lying in contact, nursing, licking and sniff/poke) observations were undertaken on 22 litters [19]. The dams were observed for the first three weeks postnatal, and then classed as high or low maternal care. By linking maternal care and temperament measured at 15–18 months old, the authors discovered a relationship between the level of maternal care given and physical engagement, social engagement and aggression. An increased interaction between puppy and dam led to adult offspring being more competitive, more engaged in social activities with humans, and with higher aggression levels (as defined by the dog’s sharpness and defence drive). The amount of maternal care given to the puppies also alters the behaviour of the puppies when they are 8-weeks-old [20]. In a similar study [20] using an isolation test, puppies that were licked more had an increased amount of exploration and a longer latency to first yelp. Increased licking also reduced the duration in locomotion and time spent interacting with the enclosure, and a shorter duration in vocalisation. These data highlight the influence that maternal care can have on future stress responses in puppies.

Currently, information regarding dam and sire selection by Australian dog breeders remains poor. The objective of this study was to understand factors considered important in the selection of Australian Purebred breeding animals with a focus on factors relating to the dam. It was expected that factors such as ANKC breed group, the number of litters produced and, whether the breed is brachycephalic would impact dam selection. The influence of sire selection and health aspects of breeding were also investigated.

## 2. Methods

### 2.1. Survey

We developed a series of questions relating to breeding practices that included questions used by Leroy et al. [4]. Breeders and ANKC members were then consulted (through email and phone) to ensure that the questions, language and terminology were appropriate for Australian dog breeders. The final survey was hosted on Qualtrics (Qualtrics, LLC, Provo, UT, USA), and was available for four months, from March to June 2015. The survey was advertised, with permission, on several online resources frequented by dog breeders. These included: Dogs SA (www.dogssa.com.au); Dogs NSW (www.dogsnsw.org.au); public breeder pages on Facebook; Dogz Online forum (www.dolforums.com.au); Vet answers blog (www.vetanswers.com.au/blog); and the German Shepherd Club of South Australia (www.gsdcsa.org.au). A description of the survey and the survey link was posted onto the researchers (VC) Facebook page and on an online community noticeboard (www.gumtree.com.au), and the survey was broadcast on local Adelaide radio (101.5 FM).

The questionnaire was anonymous and participants were not required to respond to all questions. The survey consisted of 58 questions and took approximately 15 min to complete. There were four questions pertained to the breeder’s demographics; 20 questions related to breeding management; 30 questions related to the importance of qualities associated with the dam and the sire; and four questions relating to DNA and physical testing. A full version of the questionnaire can be found in Supplementary Materials. The importance of the dam and sire were represented by multiple questions in a Likert scale where the breeder could rate the importance from Strongly Agree (1) to Strongly Disagree (5). All responses were considered for the first breed listed by the dog breeder, if the breeder bred more than one dog breed. Approval from the University of Adelaide Human Ethics Committee was obtained (H-2014-270).

### 2.2. Statistical Analysis

#### 2.2.1. Data Transformation

For open-ended responses where a range was given as a response, the average of the range was used. For example, when asked “On average, how often will you breed from each bitch?” an answer of “1–3” was then changed to “2”. To describe the physical and genetic tests performed, only those breeds with more than five respondents were included. This allowed satisfactory comparison within the data for types of dog testing. Breeders that bred more than one dog breed were excluded to remove any confusion as to which DNA or physical test was undertaken on which breed. Although some physical and genetic tests are breed specific, many times the response could be applied to several breeds (i.e., X-ray for hip score).

#### 2.2.2. Univariate Analysis of Variance (ANOVA) and PCA

Normality was not achieved in the components (components were positively skewed) and therefore they were log transformed. The Tukey method [21] was used to determine outliers. Firstly, the first and third quartile are identified. The Interquartile range (third quartile minus the first quartile) is then multiplied by 1.5. This value is subtracted from the first quartile and again added to the third quartile. Numbers which have fallen below these values are identified as outliers. Three breeders were removed as they were determined as outliers (Case numbers: 69, 180 and 223), resulting in 271 breeders for results using ANOVA. Twenty-three items relating to the dam were reduced into five components using Principal Component Analysis. However, after observing the matrix, one component was removed leaving four components. Components were analysed using a univariate general model to determine whether there were differences between Australian National Kennel Club dog breed group, number of litters produced, number of breeds the breeder owns and breeds, and brachycephalic dog breed. An eta-squared value (η^2^) was calculated; this refers to the effect size (strength between two variables) and is described for each ANOVA. Tukey post-hoc tests were used to determine significance between pairwise comparisons of means. All statistical analyses were performed using SPSS (v.21, IBM, Armonk, NY, USA). Significance was accepted at the 5% level.

## 3. Results

### 3.1. Participants

A total of 360 Australian purebred dog breeders completed the survey. However, 86 participants (24%) were discarded due to insufficient responses resulting in a total of 274 (unless stated otherwise). In particular, if any questions regarding the dog breed or dam behaviour were not answered, the respondent’s results were removed. A total of 91 dog breeds were represented in the survey, and are described in Table 1. The majority of participants bred Working dogs (21.2%) followed by Gundogs (19.3%), while the Toy group (9.9%) were the least represented (Table 1).

Brachycephalic dog breeds are those which have a facial skeleton relatively shorter than the cranial cavity [17]. These breeds included Australian Silky Terrier, Boston Terrier, Boxer, British Bulldog, Bullmastiff, Cavalier King Charles Spaniel, Chihuahua, Dogue de Bordeaux, French Bulldog, Havanese, Papillon, Pug, Rottweiler, Shar Pei, Shih Tzu, Staffordshire Bull Terrier and Tibetan Spaniel [22,23,24,25,26].

### 3.2. Principal Components Analysis Relating to the Dam

The 23 items relating to the dam were reduced using principal components analysis (PCA). Prior to performing PCA, the appropriateness of the data for analysis was assessed. The correlation matrix revealed the presence of many coefficients of 0.30 and above, the Kaiser–Meyer–Oklin exceeded the recommended value of 0.60 at 0.87, and Bartlett’s Test of Sphericity reached statistical significance, supporting the factorability of the correlation matrix. Five components were presented with an eigenvalue exceeding 1, explaining 64.06% of the total variance. After observing the rotated component matrix, four questions were removed due to either a component loading lower than 0.55 (“good cut off loading” i.e., “my dam is confident” and “my dam has an outstanding pedigree”) or too few items within the component (i.e., component 5 contained two items: “my dam has outstanding conformation according to the breed standard” and “my dam is within the accepted size according to the breed standard”) [21]. This resulted in 19 items being retained (Table 2) and four components remaining which explained 63.23% of the total variance.

The four components were labelled: Maternal Care (Component 1), Offspring Potential (Component 2), Dam Temperament (Component 3) and Dam Genetics and Health (Component 4). Component 1 was labelled Maternal Care as all questions within this component related to conception, whelping and the dam’s ability to raise and care for her puppies. Offspring Potential was labelled for Component 2 as the questions related to the offspring’s look and temperament. This Component also included the potential for temperament and genetic traits to be passed on to future generations. For example, if the dam is aggressive or rejects her puppies it may be likely that her offspring will do the same. Dam Temperament was considered for Component 3 as all questions within this component related to the dam’s behaviour. Component 4 was labelled Dam Genetics and Health as questions within this component related to health and genetically driven behaviours. The lower the score, the more the respondents found the component to be important. There were six questions related to maternal care with a range of 6–30; five questions related to offspring potential with a range of 5–25; and both genetics/health and temperament contained four questions each with a range from 4 to 20 for each component. Cronbach’s alpha for these components were 0.861, 0.747, 0.741, and 0.718 respectively.

### 3.3. General Characteristics of the Breeders

The majority of respondents were females (88%) and were aged between 18 and 85 years (*n* = 270: mean = 50.6, SD = 13.4). Most of the participants lived in New South Wales (41.6%) followed by South Australia (24.1%), Queensland (11.7%), Victoria (9.1%), Western Australia (8.8%) and Tasmania (2.6%). Six respondents (2.2%) did not report the state they were from. Half of the respondents had completed either high school (25.9%) or Technical and Further Education (TAFE) (25.9%). Some participants had completed an undergraduate degree (16.4%) with another 72 respondents (26.3%) completing a postgraduate degree. Of the remaining respondents, 5.1% had completed something other than that described, such as a diploma or a trade.

The most common dog breed within the survey was the Staffordshire bull terrier (*n* = 17, 6.2%). Participants were most likely to breed only one breed of dog (76.6%), with some participants breeding two (19.3%) or three breeds of dog (4.0%). Around one third (33.5%) had been breeding between 0 and 9 years, with the remaining having bred for more than 10 years (Table 3). Most breeders owned dams and dogs (80.3%), however 39 breeders (14.2%) only owned bitches, and 11 breeders (4.0%) only owned dogs. The remaining breeders (*n* = 4) owned neither dogs nor bitches. Almost all respondents (*n* = 267 or 97.4%) were part of the ANKC. Of the respondents who were not part of the ANKC (1.8%, *n* = 5), one breeder was associated with a working dog association recognised by the ANKC and another breeder was a member of a breed group/club.

### 3.4. Dam Breeding Priorities and ANKC Breed Group

The importance of maternal care differed significantly between ANKC breed groups (*F* (6, 264) = 2.41, *p* = 0.028; partial η^2^ = 0.05). The Toy and Hound dog breeding groups scored Maternal Care significantly more important than the Terriers, Gundogs, Working dogs and Utility groups. The Non-sporting dog breeding group scored maternal care significantly more important than the Utility breeds. Other factors were not significant for breed group: Offspring Potential (*F* (6, 264) = 1.69, *p* = 0.123; partial η^2^ = 0.04), Dam Temperament (*F* (6, 264) = 1.14, *p* = 0.341; partial η^2^ = 0.03) and Dam Genetics and Health (*F* (6, 264) = 1.59, *p* = 0.150; partial η^2^ = 0.04) (Table 4).

### 3.5. Breeding Priorities Relating to the Dam and the Number of Litters Produced

Two hundred and forty-two (89.3%) respondents bred two litters or less a year, while 29 breeders (10.7%) bred more than two litters per year. Components were not significantly different compared to the number of litters produced per year: Maternal Care (*F* (1, 267) = 3.09, *p* = 0.080; partial η^2^ = 0.07), Offspring Potential (*F* (1, 267) = 0.02, *p* = 0.892; partial η^2^ ≤ 0.01), Dam Temperament (*F* (1, 267) = 0.05, *p* = 0.817; partial η^2^ ≤ 0.01) and Dam Genetics and Health (*F* (1, 267) = 2.02, *p* = 0.156; partial η^2^ = 0.03).

### 3.6. Breeding Priorities in Relation to the Dam and the Number of Dog Breeds

Seventy-six percent of breeders bred one dog breed (*n* = 207). Dam Temperament was significantly affected by the quantity of breed types that the breeder bred (*F* (2, 268) = 3.17, *p* = 0.044; partial η^2^ = 0.07) (Table 5). The breeders of one breed type placed more importance on Dam Temperament compared to breeders who bred two types of breed. There were no significant differences between breed number and the other factors: Maternal Care (*F* (2, 268) = 1.15, *p* = 0.317; partial η^2^ = 0.03), Offspring Potential (*F* (2, 268) = 2.36, *p* = 0.097; partial η^2^ = 0.04), and Dam Genetics and Health (*F* (2, 268) = 0.91, *p* = 0.403; partial η^2^ = 0.01).

### 3.7. Breeding Priorities Relating to the Dam and Brachycephalic Dog Breeds

There were 54 breeders (19.9%) with brachycephalic dogs. Offspring Potential (*F* (1, 269) = 5.14, *p* = 0.024; partial η^2^ = 0.09) and Dam Genetics and Health (*F* (1, 269) = 4.33, *p* = 0.038; partial η^2^ = 0.06) significantly differed when comparing whether the dog breed was brachycephalic or not. Breeders of brachycephalic dogs scored Offspring Potential and Dam Genetics and Health significantly more important than breeders of non-brachycephalic dogs. There were no significant differences for non-brachycephalic and brachycephalic breeds in Maternal Care (*F* (1, 269) = 0.09, *p* = 0.771; partial η^2^ < 0.01), and Dam Temperament (*F* (1, 269) = 1.51, *p* = 0.221; partial η^2^ = 0.03) (Table 6). Seven breeders bred at least two brachycephalic dog breeds (2.6%) while 26 of the 63 breeders breeding more than one breed type bred at least one brachycephalic dog breed (9.5%).

### 3.8. Sire Selection

Twenty-nine percent of breeders accessed a distant sire owned by someone else, 23.7% of breeders used their own sire, while accessing a local sire for breeding was less common (9.9%). For breeding, the breeders’ own sire or a local sire was most commonly used (35.4%), however others imported frozen semen (6.2%) or imported the sire (0.7%). The number of breeders opting to use artificial insemination was rather small in this dataset (7.3%). Many breeders (67.8%) spent time interacting with the sire before selection but 28.0% of breeders indicated that interaction was not possible. Variables affecting sire selection are displayed in Table 7. Breeders rated the sire’s conformation and temperament highly, together with his ability to produce healthy puppies and complementing the dam.

### 3.9. Physical and Genetic Testing of Both Dams and Sires

If more than five respondents represented a single breed, they were identified for physical and genetic tests conducted. There were 11 dog breeds where this occurred (Table 8). Some breeders undertook more tests than others and many of the breeders undertook tests specific to their breed. For example, DNA testing for Neuronal Ceroid Lipofuscinosis (NCL) was undertaken by all 12 Border collie breeders while 11 out of the 14 Golden Retriever breeders undertook hip scoring.

## 4. Discussion

This study aimed to gain insight into breeding stock selection of Australian purebred dog breeders, with a particular emphasis on dams. We discuss the general characteristics and breeding priorities of a small sample of Australian purebred dog breeders covering 91 different breeds across seven breed groups classified by the ANKC. The majority of 274 breeders surveyed bred only one dog breed, kept three and two bitches and dogs respectively, and bred two litters or less a year. The implications of this study include the potential to provide the findings to dog breeding groups and governing bodies which may endorse important breeding priorities and thus produce improved dog litters.

### 4.1. The Impact of the Number of Litters Produced and the Number of Dog Breeds on Breed Priority Relating to Dams

In our sample of active and non-active breeders, almost three quarters (69%) produced one litter or less per year, which is slightly above those presented by the ANKC (54%) [3]. However, we found fewer breeders breeding 5–10 litters per year (2% compared to 5% respectively). Consistent with Leroy et al. [4] in a population of French breeders, we found that the number of litters produced was not significantly associated with breed priority. It appears that the larger kennels (determined by the number of breeding bitches and litters produced) within the current study are observant of their breeding dogs, and prioritise the health and wellbeing of their animals to a similar extent to smaller kennels. We did however, find that the breed related to the priorities and practices of the breeder. For instance, breeders of hunting and working dogs produce less stock as they are breeding to satisfy their own needs and replenish their working stock [4]. While breeders of working stock may be breeding for their own purpose, breeders of increasingly popular dog breeds, such as the Pug [27], may be producing stock for companionship and therefore litters produced would be higher.

It was expected that Dam Temperament would feature as a component from the principal component analysis given that there is a large body of work investigating temperament in dogs (e.g., [28,29,30]). Unlike the number of litters produced per year, breeders that bred a single breed rated Dam Temperament as significantly more important than breeders that bred more than one breed. Statements in this component included the dam being excitable, obedient, having a strong bond to humans, and trainable. A possible reason for the differences observed between the breeds may be due to the different dog breeds which vary in their levels of excitement, obedience and trainability. Thus, the breeder may not necessarily share the same focus across multiple breeds, which would then reduce the priority of Dam Temperament. Research into the impact of the number of breeds kept and the impact on Dam (as well as sire) Temperament is currently lacking, and needs to be investigated further.

### 4.2. Breeder Priorities in Relation to Maternal Care

Of the four breed priorities relating specifically to dams identified through PCA, questions relating to breeding and dam-puppy interactions were identified. Studies of maternal care behaviour in species such as rats, dogs and humans have shown that maternal care can have implications for the behaviour of young later in life, particularly in relation to their response to stressful events [12,13,14]. Maternal care is critical for the survival of altricial animals, where young are born immature (deaf, blind) and rely solely on their mother for survival [14,31]. Priorities relating to Maternal Care significantly differed between ANKC dog breed group, suggesting that it might be more relevant for some, but not all breeds. For instance, Maternal Care was a higher priority for breeders of Toy and Hound ANKC groups compared to the Terrier, Gundog, Working dog and Utility groups. The Maternal Care component was a mixture of statements, including conceiving and whelping with ease, as well as maternal instinct and milk production.

The majority of brachycephalic dogs consisted of breeds from the Toys and Non-sporting ANKC groups. It is common for brachycephalic dams to experience dystocia [15,16,17], difficulty of birthing the puppies naturally through the birth canal [27,32], and forcing the dam to have a caesarean birth. Bitches requiring caesareans due to dystocia account for more than 60% of births [15,33,34]. Maternal care may be impaired in dams recovering from surgery due to a caesarean birth, causing the breeder to be more involved with the litter. The dam is needed within the litter for puppy survival and development. The puppies not only feed from their mother when very young, but are also influenced by her temperament and behaviour. Further understanding of breed priorities according to the brachycephalic index would allow targeted implementation of breed standards and criteria. While comparative data on conception and prevalence of caesarean sections is lacking across all dog breeds, a recent study highlighted a general reduction in the fertility of male dogs [35]. In a retrospective cohort study of Norwegian purebred dogs, 8% of pups died before eight days of age, in 10,810 litters and 224 breeds [36]. As well as the age of the bitch and litter size, breed was also an important factor influencing perinatal mortality, although the largest variation was between litters. In breeds that experience problems in conception, birthing type of perinatal mortality, it is likely that the Maternal Care component would score more highly. For example, lack of maternal instincts may mean breeders have to work much harder to keep the puppies alive, and if early colostrum is not received by the puppies, then they will be less likely to thrive [37]. We recommend that future studies focus on questions relating specifically to maternal care and birthing method to determine the true influences on breeders’ choices in this area. An example of an important initiative in this area is the veterinary reporting of caesareans and procedures to alter the natural conformation of dogs being supported in the UK by the Kennel Club, British Veterinary Association, British Small Animal Veterinary Association and Royal College of Veterinary Surgeons [38]. Of course, we acknowledge that it is not possible to select for dam maternal behaviour until it has been observed at least once. So, although it will not influence the initial decision to breed a bitch, it should be considered for subsequent breedings.

### 4.3. Breeder Priorities in Relation to Genetics and Health

There are a large number of hereditary diseases identified in the dog population, second to humans [39]. This allows dog diseases to be identified and possibly treated in several breeds. Over 350 inherited disorders have been categorised by the American Kennel Club [7]. Of these diseases, many are restricted to specific breed groups or particular breeds [8], such as syringomyelia in the Cavalier King Charles spaniel [8]. Some diseases affect a large majority of dog breeds, e.g., hip and elbow dysplasia [40,41,42], and their heritability and incidence are continually being revised and reported. Reflective of this, breeders surveyed in this study rated Dam Genetics and Health as the most important component when selecting dams, and actively conducted genetic and physical testing of their dogs. Brachycephalic dog breeders gave even higher importance to Dam Genetics and Health than breeders of non-brachycephalic dog breeds. It is assumed that breeders of brachycephalic dogs are aware of the problems associated with these breeds of dogs (e.g., obstructive airway syndrome (BOAS) is a common constraint for brachycephalic breeds [43]), and therefore place more importance on additional genetic and health aspects which may also be present. Genetic testing was more relevant to some breeds than others, likely reflecting known issues within the breed. For example, all of the Rottweiler breeders assessed their dogs for dentition assessment, indicating that this is an important problem for the breed. Eight breeds were assessed for elbow scoring. Indeed, elbow dysplasia and borderline signs were observed in half of the Rottweilers included in an official screening program (see [44]).

Important diseases have been recorded in dogs and by all breeders (http://discoveryspace.upei.ca/cidd/breeds/overview). A review of common disorders inherited in purebred dogs is also available [18]. In purebred dogs, in a case-control study for 24 common hereditary disorders, 10 disorders were found to be more prevalent than in mixed-breed dogs, suggesting a greater proportion of diseases occurring in purebred than in mixed-breed dogs [45]. Multiple disorders are associated with brachycephalic breeds: BOAS in Boston Terriers and Pugs [46], dystocia in Bull Terriers [27], eye problems in Pugs and Shar Peis, and mitral valve disease in Cavalier King Charles Spaniels [27]. Only one breeder of six described undertaking a breathing test for their pug, although 67% (*n* = 6) documented eye testing. While no common tests were undertaken by Boston Terrier (*n* = 2) and Shar Pei breeders (*n* = 1), almost all breeders of Cavalier Kind Charles Spaniels (6/7) had hear auscultations as a physical test undertaken on their dogs. It seems important to highlight that a common disease test for the Pug is not being undertaken, and needs further investigation as to the reasons behind this. Although Dam Genetics and Health were deemed more important by brachycephalic breeders, it seems that they are not always undertaking the relevant tests. In the current dataset, all Great Dane breeders tested for dilated cardiomyopathy and almost all of Rottweiler breeders tested for elbow dysplasia. The top ten significant disorders of the ANKC include hip dysplasia, epilepsy, hypothyroidism, allergies, hemangiosarcoma, patella luxation, cataracts, lymphoma, bloat and progressive retinal atrophy [47]. Of these important diseases listed by the ANKC, all of the Great Dane breeders tested for hip dysplasia. However, even though hip dysplasia is a common problem in Labrador Retrievers and German Shepherds [48], only around half of breeders reported using Hip Scores on their breeding dogs. This may have been due to not all breeders completing this section of the survey, as registered breeders of Labrador Retrievers must have their breeding dogs hip scored, although this is not mandatory for German Shepherd breeders in Australia [49,50]. Further investigation into the reasons behind decisions to undertake genetic and health testing are required. Such knowledge is likely to help the efforts to increase the number of breeders undertaking tests on their breed stock.

### 4.4. Sire Selection

Only a small portion of the breeders surveyed used their own sires. When seeking sires, breeders preferred “distant” over “local” sires, and where possible, still preferred to meet and interact with the sire. Travelling distances to find sires might be required to ensure genetic diversity, which is particularly important for closed breeding lines. The location of the sire was a low priority for breeders, but conformation, size, pedigree, temperament, that the sire complements the dam, and the sire produces healthy puppies were all considered important in their decision. Only a small percentage chose to use artificial insemination methods. There are both advantages and disadvantages of artificial insemination (fresh or frozen semen) and natural mating in dogs. Advantages include an increased level of hygiene [9] and safety, as well as long term storage and the high number of usage per sire [51]. Natural mating in dogs may result in reduced whelping rates compared to artificial insemination using frozen semen [10]. Disadvantages of artificial insemination exist and include a limited shelf life, expensive to store and disruption of sperm numbers [51]. Artificial insemination using frozen semen results in a smaller litter size compared to artificial selection using fresh semen [52,53]. Artificial insemination may lessen the likelihood that the dam would become sick or injured during impregnation, allowing a more successful pregnancy due to reduced stress. If not already, details surrounding artificial means should be discussed between breeders and veterinarians given the animal welfare implications for the bitch.

### 4.5. Limitations and Future Work

There were a number of limitations of the survey, particularly the low number of breeders who participated. Given the nature of the survey where participants self-selected, non-response bias cannot be calculated however, the results may be representing participants who were responsible and conscientious breeders registered with the ANKC. Thus, the findings reported here do not necessarily reflect the priorities or practices or all purebred breeders across Australia, nor represent all of the different breeds of dogs. It is equally important to understand and compare the practices of non-registered Australian dog breeders [54] as they are likely to differ in their breeding practices.

A number of important questions were unintentionally omitted from this study. For example, future studies should ask for the number of puppies born alive per year, whether it was more likely for the dam/(s) to give birth naturally or by caesarean, and other factors which may be important for specific breeds (i.e., hunting/retrieving capabilities for gundogs). Some issues were discovered with several questions that could also be improved for future studies. For example, our questions relating to priorities and practices were not breed specific, which did not allow breeders that bred more than one breed to be distinguished. In relation to accessing sires, there was some confusion as to the definition of *distant sire* given what people constitute as “distant”, and their willingness to travel (or import semen) are likely to vary among breeders and especially breeders of rare breeds. The survey also highlighted that some breeders wanted to undertake physical or DNA testing for their dogs but there were reasons as to why this was not conducted. For some, it was not physically possible (i.e., the breeder was in a remote location), tests were unavailable within Australia or there were no specific tests available. The expense of such testing may also influence decisions. We recommend that those involved with dog breeding and breed maintenance (i.e., veterinarians, ANKC) discuss options that are available to breeders so such tests can be accessed.

## 5. Conclusions

This study represents a step toward understanding breeding stock selection and breed priorities of Australian purebred dog breeders. Emphasis was given to breeding priorities and practices surrounding the Dam, given the important role maternal care has on the development of puppies. These findings provide useful insight into dog breeding, and provide information that may be helpful for dog breeding groups and governing bodies (such as state governments, the ANKC) to manage breeding and breeder education. For example, the impact of caesarean births on mothering ability could be addressed to highlight the importance the mother has on puppy health and behaviour throughout the puppy’s life. A significant association between ANKC breed group, the number of dogs the kennel bred, and whether the breed was brachycephalic on breed priority was found. This suggests that a “one size fits all” model for selecting and managing breeding stock is not appropriate. Importantly, it seemed that many breeders did not prioritise the maternal care behaviour of the dams. Emphasis of maternal care as a selection factor (for subsequent breeding of a bitch) should be made more prominent to breeders due to the impact it may have on the puppy’s stress-related behaviour later in life.

## Figures and Tables

**Table 1:** Dog breeds represented in the survey.

**Group 1: Toys**	**N**	**%**	**Group 2: Terriers**	**N**	**%**	**Group 3: Gundogs**	**N**	**%**	**Group 4: Hounds**	**N**	**%**
Australian Silky Terrier *	2	0.7	American Staffordshire Terrier	1	0.4	Brittany	1	0.4	Afghan Hound	2	0.7
Cavalier King Charles Spaniel *	4	1.5	Bull Terrier	2	0.7	Cocker Spaniel	8	2.9	Basenji	5	1.8
Chihuahua *	1	0.4	Bull Terrier Miniature	1	0.4	Field Spaniel	2	0.7	Beagle	3	1.1
Chinese Crested Dog	1	0.4	Jack Russell Terrier	2	0.7	Flat Coated Retriever	1	0.4	Borzoi	1	0.4
Havanese *	2	0.7	Scottish Terrier	2	0.7	German Shorthaired Pointer	5	1.8	Dachshund (Min. Long)	1	0.4
Italian Greyhound	6	2.2	Soft Coated Wheaten Terrier	1	0.4	Golden Retriever	15	5.5	Dachshund (Min. Smooth)	1	0.4
Miniature Pinscher	2	0.7	St. Bernard	1	0.4	Gordon Setter	1	0.4	Deerhound	1	0.4
Papillon *	2	0.7	Staffordshire Bull Terrier *	17	6.2	Hungarian Vizsla	1	0.4	Petit Basset Griffon Vendeen	1	0.4
Pug *	5	1.8	Tenterfield Terrier	1	0.4	Hungarian Wirehaired Vizsla	1	0.4	Rhodesian Ridgeback	3	1.1
Tibetan Spaniel *	2	0.7	West Highland White Terrier	5	1.8	Labrador Retriever	12	4.4	Saluki	4	1.5
						Nova Scotia Duck Tolling Retriever	3	1.1	Whippet	6	2.2
						Weimaraner	2	0.7			
						Welsh Springer Spaniel	1	0.4			
Total Group 1	27	9.9	Total Group 2	33	12.0	Total Group 3	53	19.3	Total Group 4	28	10.2
**Group 5: Working Dogs**	**N**	**%**	**Group 6: Utility**		**N**		**%**		**Group 7: Non-Sporting**	**N**	**%**
Australian Cattle Dog	1	0.4	Alaskan Malamute		3		1.1		Boston Terrier *	1	0.4
Australian Kelpie	6	2.2	Boxer		6		2.2		British Bulldog	2	0.7
Australian Shepherd	6	2.2	Bullmastiff		2		0.7		Dalmatian	1	0.4
Belgian Shepherd Dog	7	2.6	Cane Corso		2		0.7		French Bulldog *	1	0.4
Border Collie	13		Dobermann		2		0.7		German Spitz	1	0.4
Collie (Rough)	2	0.7	Dogue de Bordeaux *		1		0.4		Great Dane	7	2.6
Collie (Smooth)	1	0.4	German Pinscher		1		0.4		Japanese Spitz	1	0.4
Finnish Lapphund	2	0.7	Leonberger		1		0.4		Poodle (Miniature)	3	1.1
German Shepherd Dog	13	4.7	Neapolitan Mastiff		1		0.4		Poodle (Standard)	7	2.6
Maremma Sheepdog	1	0.4	Newfoundland		2		0.7		Poodle (Toy)	2	0.7
Shetland Sheepdog	2	0.7	Old English Sheepdog		2		0.7		Schipperke	1	0.4
Welsh Corgi (Cardigan)	1	0.4	Portugese Water Dog		1		0.4		Shar Pei *	1	0.4
Welsh Corgi (Pembroke)	3	1.1	Pyrenean Mountain Dog		1		0.4		Shih Tzu *	1	0.4
			Rottweiler *		10		3.6		Tibetan Terrier	1	0.4
			Russian Black Terrier		2		0.7		Xoloitzcuintle	2	0.7
			Samoyed		1		0.4				
			Schnauzer		1		0.4				
			Siberian Husky		3		1.1				
			Tibetan Mastiff		1		0.4				
Total Group 5	58	21.2	Total Group 6		43		15.7		Total Group 7	32	11.7

* Brachycephalic dog breed (Brachycephalic dog breeds are those which have a facial skeleton relatively shorter than the cranial cavity [17]).

**Table 2:** Loading for the 19 items of the dam scales generated by means of Principal Component Analysis.

	Component			
Survey Question	1	2	3	4
My dam has naturally conceived with ease	0.733			
My dam has whelped with ease	0.803			
My dam has an excellent maternal instinct towards her puppies	0.729			
My dam produces sufficient milk to raise her puppies	0.762			
I would not breed from a dam if conception and whelping were difficult	0.675			
I would not breed from a dam if her maternal behaviour was not ideal	0.625			
I would not breed from a dam if her puppies did not conform closely to the breed standard		0.705		
I would not breed from a dam if the temperament of her puppies was not ideal		0.761		
I would not breed from a dam if she had rejected her puppies		0.567		
I would not breed from a dam if some of her puppies had a significant genetic fault		0.735		
I would not breed from a dam that was aggressive towards unknown people		0.605		
My dam is excitable			0.617	
My dam is obedient			0.806	
My dam has a strong bond to humans			0.659	
My dam is trainable			0.761	
My dam has an optimal temperament for the breed				0.747
My dam has passed all required and recommended health tests for her breed				0.735
My dam comes from a line of healthy, long lived relatives				0.658
My dam is friendly to dogs, other animals and people				0.604

**Table 3:** Number of years dog breeders had bred and/or owned their dogs.

	No. of Years Breeding Dog		No. of Years Owning Dog	
Years	N	%	N	%
<5 years	59	21.5	13	4.7
5–9 years	33	12.0	38	13.9
10–19 years	65	23.7	73	26.6
20–29 years	51	18.6	70	25.5
30–39 years	32	11.7	53	19.3
40+ years	33	12.0	27	9.9

**Table 4:** Principal Component Analysis subscale relating to the Dam according to ANKC breed group. Lower values represent higher importance.

						95% Confidence Interval	
Outcome Variable	df	Df Error	*F*	Breed Group	Means	Lower Bound	Upper Bound
Maternal care	6	911.73	2.408	Toys	0.916	0.858	0.974
				Terriers	0.986	0.934	1.038
				Gundogs	0.977	0.937	1.018
				Hounds	0.906	0.850	0.962
				Working dogs	1.000	0.962	1.038
				Utility	1.011	0.965	1.057
				Non-Sporting	0.959	0.907	1.011
Offspring Potential	6	911.73	1.693	Toys	0.818	0.767	0.868
				Terriers	0.860	0.814	0.905
				Gundogs	0.855	0.820	0.891
				Hounds	0.827	0.778	0.875
				Working dogs	0.893	0.859	0.926
				Utility	0.845	0.805	0.885
				Non-Sporting	0.825	0.780	0.871
Dam Temperament	6	911.73	1.138	Toys	0.913	0.857	0.969
				Terriers	0.929	0.878	0.979
				Gundogs	0.923	0.884	0.962
				Hounds	0.993	0.939	1.047
				Working dogs	0.937	0.899	0.974
				Utility	0.964	0.919	1.008
				Non-Sporting	0.934	0.883	0.984
Dam Genetics and Health	6	911.73	1.591	Toys	0.674	0.631	0.718
				Terriers	0.702	0.663	0.742
				Gundogs	0.678	0.647	0.708
				Hounds	0.747	0.705	0.789
				Working dogs	0.704	0.675	0.733
				Utility	0.691	0.657	0.726
				Non-Sporting	0.675	0.636	0.715

**Table 5:** Breeder priorities relating to the Dam and the number of breeds that the participant bred.

						95% Confidence Interval	
Outcome Variable	df	Df Error	*F*	Number of Breeds	Means	Lower Bound	Upper Bound
Maternal care	2	530	1.154	1	0.965	0.945	0.986
				2	1.000	0.959	1.042
				3	0.988	0.894	1.083
Offspring	2	530	2.357	1	0.847	0.829	0.864
				2	0.859	0.823	0.894
				3	0.937	0.856	1.018
Dam Temperament	2	530	3.168	1	0.929	0.909	0.949
				2	0.985	0.946	1.024
				3	0.946	0.857	1.036
Dam Genetics and health	2	530	0.911	1	0.691	0.675	0.706
				2	0.704	0.673	0.735
				3	0.734	0.664	0.805

**Table 6:** Breed priority according to whether the dog breed was brachycephalic.

						95% Confidence Interval	
Outcome Variable	df	Df Error	*F*	Brachycephalic Breed	Means	Lower Bound	Upper Bound
Maternal care	1	266	0.085	No	0.974	0.954	0.995
				Yes	0.968	0.927	1.008
Offspring	1	266	5.138	No	0.861	0.844	0.879
				Yes	0.816	0.781	0.851
Dam Temperament	1	266	1.505	No	0.946	0.926	0.965
				Yes	0.919	0.880	0.958
Dam Genetics and health	1	266	4.326	No	0.702	0.687	0.717
				Yes	0.666	0.636	0.697

**Table 7:** Variables important to the selection of the sire.

	Respondents (%)						
Variable Importance	Location	Conformation	Size	Pedigree	Temperament	Complements Dam	Healthy Puppies
High	6.0	95.5	84.7	74.3	98.9	99.6	95.5
Neutral	10.9	3.7	14.6	20.5	1.1	0.0	3.7
Low	83.0	0.7	0.7	5.2	0.0	0.4	0.7

**Table 8:** Genetic and Physical testing of common dog breeds within the survey.

Breed	Breeders (*n*)	DNA Test	Breeders Undertaking Test (*n*)	Physical Tests	Breeders Undertaking Test (*n*)
Basenji	5	Fanconi	5	Eye assessment	5
		Progressive Retinal Atrophy	4	Hip score	2
		Hemolytic anaemia	2	Thyroid	2
		Pyruvate kinase deficiency	1	Heart assessment	1
		DNA inbreeding coefficient Factor 7	1		
		DNA identification Thyroid	1		
Belgian Shepherd Dog	5	Colour MDR1 masking	1	Hip score	3
				Elbow score	*3*
				Eye assessment	*2*
				Heart assessment	*1*
Border Collie	12	Neuronal Ceroid Lipofuscinosis	12	Elbow score	11
		Trapped Neutrophil Syndrome	11	Hip score	10
		Collie Eye Anomaly	10	Eye assessment	6
		Multi-Drug Resistance Gene 1	2	General vet check	1
		Imerslund-Grasbeck Syndrome	2	Chiropractor vet check	1
		Degenerative Myelopathy	1	Collie collapse	1
		Parentage (Orivet)	1	Hearing test	1
		Glaucoma	1		
		B12	1		
German Shepherd Dog	11	Degenerative Myelopathy	6	Hip score	6
		Ivermectin Sensitivity	2	Elbow score	6
		Long stock coat gene	1	X-ray (not specified)	1
		Canine renal Dysplasia	1	Vet check	1
		Dwarfism	1		
		Haemophilia	1		
Golden Retriever	14	Ichthyosis	12	Hip score	11
		Progressive Retinal Atrophy 1	10	Eye assessment	11
		Progressive Retinal Atrophy 2	10	Heart assessment	11
		Progressive Rod Cone Degeneration	4	Elbow score	10
				Dentition assessment	1
Great Dane	5	Heart testing	5	Hip score	5
		Thyroid	4	Elbow score	5
		Colour DNA	1	Shoulder and neck X-rays	1
Labrador Retriever	11	Progressive Retinal Atrophy	10	Hip score	5
		Exercise-induced Collapse	8	Elbow score	5
		Progressive Rod Cone Degeneration	2		
		Coat colour	1		
		Long hair	1		
		DNA identification	1		
		Centronuclear Myopathy	1		
Poodle (Standard)	5	Degenerative Myelopathy	3	Eye assessment	4
		Neonatal Encephalopathy	3	Hip score	4
		von Willebrand’s disease	3	Skin biopsy	1
		Thyroid	2	Vet checked	1
		Full DNA data	1		
		Progressive Retinal Atrophy	1		
		Renal Dysplasia	1		
Rottweiler	8	DNA testing	1	Hip score	8
		von Willebrand’s disease	1	Eye assessment	8
				Dentition assessment	8
				Elbow score	7
				Heart assessment	3
				Joint assessment	1
				Brachycephalic Obstructive Airway Syndrome	1
Staffordshire Bull Terrier	16	Hereditary Cataracts	12	Eye assessment	7
		L2-Hydroxyglutaric aciduria	12	Hip score	3
		Full DNA test	2	Heart assessment	2
		Coat colour	1	Elbow score	1
				Dentition assessment	1
				X-ray (not specified)	1
				Vet check	1
West Highland White Terrier	5	Genetic technologies	1		

## Supplementary Materials

The following are available online at www.mdpi.com/2076-2615/6/11/75/s1, Breeder Survey questions.
