# Supplementary material for: Selection of Breeding Stock among Australian Purebred Dog Breeders, with Particular Emphasis on the Dam
Source: Animals (Basel). 2016 Nov 16;6(11):75. doi: 10.3390/ani6110075 (PMC5126777; doi:10.3390/ani6110075)
Supplement: Supplementary file 1 [file animals-06-00075-s001.pdf]

Supplementary Materials: Selection of breeding stock among Australian purebred dog breeders, with particular emphasis on the dam

Veronika Czerwinski, Michelle McArthur, Bradley Smith, Philip Hynd and Susan Hazel

Breeder Survey questions

Breeder Choices Survey

(Please circle your answers)

About you

1. Gender: Male Female

2. Age: ________

3. Postcode: ________

4. Highest level of education:

High School TAFE Undergraduate Post-graduate
Other? ______________

5. What breed (/s) of dog (/s) do you breed?

____
____
____

6. How long have you owned your breed/s?

____
____
____

7. How long have you bred dogs for?

_________ years

8. How many litters of puppies do you normally breed per year?

_________litters

9. Do you show dogs?

Never Seldom Often

10. Do you participate in other ANKC recognised competitive dog sports?
    Never Seldom Often

11. Are you a member of your state or territory canine council, which is a member of body of the Australian National Kennel Council (ANKC)?

Yes No

12. Are you a member of an ANKC recognised working dog association?
    Yes No

13. Are you a member of a breed club, or a group if no breed club exists?

Yes No

14. How many bitches do you currently own that you breed from or hope to breed from in the future?

____

15. How many dogs (male and female) in total do you currently own?
    _______dogs &______bitches

16. Where do you advertise the puppies when they are ready to be sold? *Please tick as many as applicable.*

☐Gumtree ☐Trading Post ☐DogzOnline
☐Other website (please list) _______________ ☐ Word of mouth
☐Newspaper ☐Other (please list)_________________________

About the dam (/s)

17. On average, how often will you breed from each bitch?

_________ times in their life

18. At what age do you usually first breed from your bitches?

____

19. At what age do you usually last breed from your bitches?
    ____

20. Do some of your bitches usually live with somebody else?
    Yes No

21. Where do your bitches usually live?

Outdoors Indoors Both

22. How important are the following in your decision to breed from an ideal dam?
Please tick once for each statement

	Strongly disagree	Disagree	Neither agree nor disagree	Agree	Strongly agree
My ideal dam has outstanding conformation according to the breed standard					
My ideal dam is within the accepted size according to the breed standard					
My ideal dam has an optimal temperament for the breed					
My ideal dam has passed all required and recommended health tests for her breed					
My ideal dam comes from a line of healthy, long lived relatives					
My ideal dam is friendly to dogs, other animals and people					
My ideal dam is excitable					
My ideal dam is obedient					
My ideal dam is confident					
My ideal dam has a strong bond to humans					
My ideal dam is trainable					
My ideal dam has an outstanding pedigree					
My ideal dam has naturally conceived with ease					
My ideal dam has whelped with ease					
My ideal dam has an excellent maternal instinct towards her puppies					
My ideal dam produces sufficient milk to raise her puppies					

23. Are tests available for genetic disorders within your breed/s?

Yes No

24. Do you undertake DNA testing of your breeding animals for any genetic disorders? If yes, which ones?

25. Do you undertake physical testing for genetic disorders, such as heart and eye examinations? If yes, which ones?

26. How important are the following in your decision to remove a dam from your breeding stock?

Please tick once for each statement

	Strongly disagree	Disagree	Neither agree nor disagree	Agree	Strongly agree
I would not breed from a dam if conception and whelping were difficult					
I would not breed from a dam if her maternal behaviour was not ideal					

I would not breed from a dam if her puppies did not conform closely to the breed standard					
I would not breed from a dam if the temperament of her puppies was not ideal					
I would not breed from a dam if she had rejected her puppies					
I would not breed from a dam if some of her puppies had a significant genetic fault					
I would not breed from a dam if she was aggressive towards strangers					

About the sire

27. Where are you most likely to access a sire?

Own sire Local sire owned by someone else
Distant sire owned by someone else

28. If the sire is sourced from elsewhere (not own sire), do you personally spend time with the sire observing his temperament and structure before mating him to your bitch (/es)?

Yes No Not possible

29. Rate the following factors for their importance in choosing a sire to breed to your dam?

Please tick once for each factor

	Strongly disagree	Disagree	Neither agree nor disagree	Agree	Strongly agree
My ideal sire lives conveniently close to me					
My ideal sire has outstanding conformation according to the breed standard					
My ideal sire is the accepted size according to the breed standard					
My ideal sire has an outstanding pedigree (desired ancestors in common with the dam)					
My ideal sire has an optimal temperament for the breed					
My ideal sire complements my bitch's virtues and does not share her faults					
My ideal sire has produced puppies which are healthy and conform closely to the breed standards when mated to other bitches					

30. How important is the coefficient of inbreeding (COI) of the litter when determining which bitch and dog will be mated? *Please circle one*

Very important Slightly important Neutral Slightly unimportant Not important
I do not know what COI is

Other information

31. Is there any further information you would like to share regarding your breeding animals or breeding practices?

Thank you for completing this survey!

© 2016 by the authors. Submitted for possible open access publication under the terms and conditions of the Creative Commons Attribution (CC-BY) license (http://creativecommons.org/licenses/by/4.0/).
